# Exome Sequencing of Index Patients with Retinal Dystrophies as a Tool for Molecular Diagnosis

**DOI:** 10.1371/journal.pone.0065574

**Published:** 2013-06-14

**Authors:** Marta Corton, Koji M. Nishiguchi, Almudena Avila-Fernández, Konstantinos Nikopoulos, Rosa Riveiro-Alvarez, Sorina D. Tatu, Carmen Ayuso, Carlo Rivolta

**Affiliations:** 1 Department of Genetics, IIS- Fundacion Jimenez Diaz, CIBERER, Madrid, Spain; 2 Department of Medical Genetics, University of Lausanne, Lausanne, Switzerland; Justus-Liebig-University Giessen, Germany

## Abstract

**Background:**

Retinal dystrophies (RD) are a group of hereditary diseases that lead to debilitating visual impairment and are usually transmitted as a Mendelian trait. Pathogenic mutations can occur in any of the 100 or more disease genes identified so far, making molecular diagnosis a rather laborious process. In this work we explored the use of whole exome sequencing (WES) as a tool for identification of RD mutations, with the aim of assessing its applicability in a diagnostic context.

**Methodology/Principal Findings:**

We ascertained 12 Spanish families with seemingly recessive RD. All of the index patients underwent mutational pre-screening by chip-based sequence hybridization and resulted to be negative for known RD mutations. With the exception of one pedigree, to simulate a standard diagnostic scenario we processed by WES only the DNA from the index patient of each family, followed by *in silico* data analysis. We successfully identified causative mutations in patients from 10 different families, which were later verified by Sanger sequencing and co-segregation analyses. Specifically, we detected pathogenic DNA variants (∼50% novel mutations) in the genes *RP1*, *USH2A*, *CNGB3, NMNAT1*, *CHM*, and *ABCA4*, responsible for retinitis pigmentosa, Usher syndrome, achromatopsia, Leber congenital amaurosis, choroideremia, or recessive Stargardt/cone-rod dystrophy cases.

**Conclusions/Significance:**

Despite the absence of genetic information from other family members that could help excluding nonpathogenic DNA variants, we could detect causative mutations in a variety of genes known to represent a wide spectrum of clinical phenotypes in 83% of the patients analyzed. Considering the constant drop in costs for human exome sequencing and the relative simplicity of the analyses made, this technique could represent a valuable tool for molecular diagnostics or genetic research, even in cases for which no genotypes from family members are available.

## Introduction

Retinal dystrophies (RD), comprising the wide spectrum of retinal degeneration, are rare genetic conditions leading to visual deficiency and in some instances to blindness [1]. These diseases affect roughly one person out of 4,000 and are the result of the progressive loss or dysfunction of photoreceptors, the light-sensing cells of the eye [2]. RD are caused by mutations in more than 100 different genes (RetNet; https://sph.uth.edu/Retnet/home.htm). Mutations causing RD can occur in any of these genes, as well as in other disease genes that still await identification, and can be transmitted as dominant, recessive, or X-linked alleles. This very elevated genetic heterogeneity is possibly the highest detected so far among all Mendelian diseases and leads to a very high carrier frequency of heterozygous mutant alleles, i.e. possibly more than 1 in 4–5 individuals [3]. Molecular diagnosis is therefore an extremely daunting task since unrelated patients with the similar clinical presentations are likely to have defects in different genes, each of which with a small chance to be found positive for mutations. In other cases, accurate molecular diagnosis is hampered by limited clinical information, which is critical for efficiently deciding which genes have to be analyzed. To bypass the drawbacks of classical exon-by-exon PCR screenings, associated with very long processing times, the use of mutation- or gene-specific microarrays has been recently adopted [4]–[12], with different results reported by us and others to be largely dependent on the characteristics of the population screened [13]–[19].

Whole exome sequencing (WES) is a procedure that allows the purification by sequence capture of all exonic regions of a genome and their further processing by next-generation sequencing (NGS) [20]. Since the selection of the target regions to be sequenced by WES spans all of the genome, this method is not conventionally used to interrogate a specific DNA fragment or a limited number of genes, but conversely it is adopted to investigate genetic features at a genome scale. Moreover, in virtue of their general applicability, WES protocols and kits have diffused rapidly and have become more and more affordable, to the point of being in some instances less expensive than targeted DNA capture/NGS projects interrogating a lower number of DNA features.

In this work, we evaluate the possibility of using WES as a tool for routine molecular diagnoses in patients with apparently recessive RD. Although we specifically selected families with multiple affected members to allow validation of the findings, we willingly ignored the information related to the pedigree, to simulate the use of this technique for the large majority of people with RD, i.e. isolate patients.

## Materials and Methods

### Ethics Statement

This study was carried out in accordance with the tenets of the Declaration of Helsinki and was approved by the Institutional Review Boards of the University of Lausanne and the Clinical Research Ethics Committee of the Fundacion Jimenez Diaz. Written informed consent for WES analyses was obtained from the subjects who participated in this study and donated their blood for research. Each individual was anonymized by assigning to him/her a numeric ID; confidentiality and protection of data were ensured by applying international recommendations and current Spanish legislation (Ley de Investigacion Biomedica 14/2007 and LOPD).

### Patients

All patients were previously tested and all resulted to be negative for known autosomal recessive retinitis pigmentosa (ARRP) or Leber Congenital Amaurosis (LCA) mutations by microarray screening, based on the Arrayed Primer EXtension (APEX) technology [Asper Ophthalmics, Tartu, Estonia] [21]. Seven families (RP-0137, RP-0298, RP-0461, RP-1102, RP-1116, RP-1164, RP-1263) were also analyzed by whole genome homozygosity mapping using SNP arrays from Affymetrix (Genome Wide Human SNP array 6.0 and GeneChip Human Mapping 500K Array Set) or Illumina (HumanLinkage V Panel Set or Omni Whole Genome arrays HumanCytoSNP-12), as previously described [22]. Affymetrix genotyping services were provided by the Spanish “Centro Nacional de Genotipado” (CEGEN-ISCIII). No significant homozygous regions larger than 1 Mb were found in these families. Only index patients from each family were analyzed by WES, except for family RP-0235, for which all 5 members underwent WES analyses.

### Sample Preparation

Genomic DNA was extracted from 7 ml of whole blood using an automated DNA extractor (BioRobot EZ1 Qiagen, Hilden, Germany) following the manufacturer’s instructions.

### Library Preparation and Sequencing

Purification of target sequences, library preparation, and NGS processing was performed by BGI-Shenzen (Shenzhen, China), as previously described [23].

### Alignment and Analysis of Reads

Reads were mapped to the human genome reference sequence (NCBI build 36.1) and call of variants was performed according to previously published algorithms and procedures [24]. These procedures were performed by BGI, under a fee-for-service agreement.

### Filtering Procedures of Detected Variants

DNA variants identified following mapping and evaluation of the reads were assessed to represent actual RD mutations by a 5-step filtering procedure, mainly by the use of simple Perl scripts developed in house and available upon request and/or by the use of standard electronic spreadsheets. First, all variants that were not part of coding sequences or represented silent changes were eliminated, to produce a set of DNA changes that would include only missense, nonsense, and indel events, as well as substitutions affecting exon splicing signals. A second step consisted of selecting only variations found to affect 160 known RD genes. The number of remaining variants was then further reduced by removing known SNPs that were present in dbSNP version 130 (a version that is devoid of data from large-scale endeavors) and having an allelic frequency higher than 0.02. Subsequently, a fourth filtering procedures consisted in ascertaining RD genes carrying two of these variants (or the same variant, but homozygously) to account for the recessive mode of inheritance. At this point, all candidate variants were carefully checked for previous description in the literature and databases, including dbSNP version 137.

### PCR and Sanger Sequencing

Variants detected by NGS and suspected to be pathogenic were re-amplified by PCR by using as a template an aliquot of the same DNA samples that were used for WES. Cycling conditions and primers used are listed in Table S1. PCR products were enzymatically purified using ExoSAP-it (USB, Affymetrix, Santa Clara, CA) and sequenced on both strands using the Big Dye Terminator Cycle Sequencing Kit v3.1 Kit (Applied Biosystems). The sequence products were purified on a 96-well multiscreen filter plate (Montage SEQ96 Sequencing Reaction Cleanup Kit, Millipore, Bedford, MA) and resolved on a 3130xl ABI instrument (Applied Biosystems). Chromatograms were interpreted and aligned to the human reference sequence using the STADEN package [25].

## Results

### Ascertainment of Patients

Twelve Spanish families with recessive RD were ascertained. Diagnoses were based on ophthalmologic examination and pedigree data, according to previously described/established clinical and classification criteria [26]–[30]. Clinical findings of these patients are summarized in Table S2.

### Detection and Filtering of DNA Variants

The number of variants remaining after the application of each of the five filtering processes is summarized in Table S3. In short, we detected on average 67,000 DNA variants per genome. Of these, only ∼12,000 represented changes that could potentially alter the sequence or the structure of coding transcripts. Among them, 108 to 143 variants were present in the 160 genes that were previously found to cause RD and 18 to 34 were rare changes (less than 2% in frequency) or were not registered in dbSNP 130. The few variants that were identified as 2 alleles (either as a compound heterozygote with another variant or as a homozygote) in a single RD gene and thus could account for the recessive inheritance pattern were further analyzed.

### Detection of Mutations in Known RD Genes

**Figure 1:**
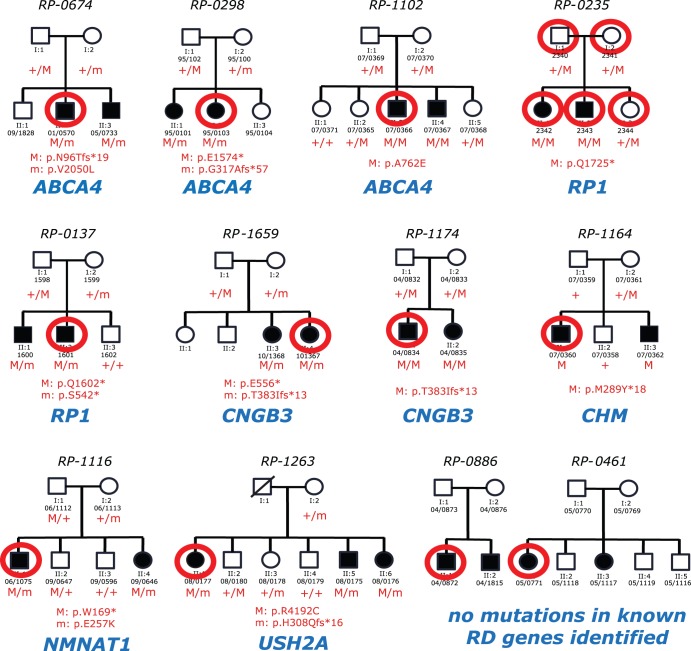
Pedigrees of patients analyzed and mutations identified in this work. The family ID is given above the pedigree, while the individuals’ IDs are indicated below the symbols depicting them. Red circles indicate individuals whose DNA underwent WES analysis. The name of the RD gene identified as causative of the disease is given in blue. M/M, homozygous mutation; M/m compound heterozygous mutations.

Ten of the 12 index patients analyzed by WES were found to be either homozygous or compound heterozygous for variants in known RD genes that would satisfy our filtering criteria (Table 1, Figure 1).

**Table 1:** RD mutations identified by WES analyses.

FAMILY ID	INDEX PATIENT ID	GENE (OMIM entry)	NUCLEOTIDE CHANGE	PROTEIN CHANGE	NOVEL/KNOWN	REFERENCE
RP-0674	01-0570	*ABCA4*	c.287delA	p.N96Tfs*19	novel	
		(601691)	c.6148G>C	p.V2050L	known	[43]
RP-0298	95-0103	*ABCA4*	c.4720G>T	p.E1574*	known	[44]
			c.950delG	p.G317Afs*57	novel	
RP-1102	07-0366	*ABCA4*	c.2285C>A (homoz)	p.A762E	known	[45]
RP-1164	07-0360	*CHM*(300390)	c.863dupA	p.M289Y*18	novel	
RP-1263	08-0177	*USH2A*	c.920_923dupGCCA	p.H308Qfs*16	known	[46]
		(608400)	c.12574C>T	p.R4192C	novel	
RP-1659	10-1367	*CNGB3*	c.1148delC	p.T383Ifs*13	known	[31]
		(605080)	c.1666G>T	p.E556*	novel	
RP-1174	04-0834	*CNGB3*	c.1148delC (homoz)	p.T383Ifs*13	known	[31]
RP-0137	1601	*RP1*	c.1625C>G	p.S542*	novel	
		(603937)	c.4804C>T	p.Q1602*	novel	
RP-0235	2343	*RP1*	c.5173C>T (homoz)	p.Q1725*	novel	
RP-1116	06-1075	*NMNAT1*	c.507G>A	p.W169*	known	[47]
		(608700)	c.769G>A	p.E257K	known	[47]

More specifically, 3 patients/families were positive for mutations in *ABCA4*, 2 had mutations in the *RP1* gene, and 2 others in the *CNGB3* gene. The remainder of these carried variants in *CHM*, *USH2A*, and *NMNAT1*. All of these mutations cosegregated perfectly with the disease in all families, according to a recessive or, in the case of family RP-1164, X-linked pattern of inheritance, as ascertained by exon-PCR and Sanger sequencing (Figure 1). Of the 15 different mutations identified, 8 were never described previously and included 7 changes that were clearly deleterious alleles (frameshifts or nonsenses, Table 1). The remaining mutation was a missense change (c.12574C>T, p.R4192C) identified in the *USH2A* gene. This was considered pathogenic in conjunction with another clear-cut mutation (c.920_923dupGCCA, p.H308Qfs*16) based on the following reasons. First, cosegregation analysis in the mother and 5 other siblings (2 affected and 3 unaffected) was statistically significant, with a p-value of 0.006 (Figure 1). Second, the mutation was not found in 100 ethnically-matched healthy controls or any other public database, including the one from the 1000 Genomes Project. Lastly, *in silico* assessment of the consequence of the missense mutation predicted the change to affect the function of the translated protein (SIFT and Polyphen).

All patients identified with disease-associated mutations had no other genes/variants that fulfilled our filtering criteria, except for individual 04/0834. This patient carried the previously-reported homozygous *CNGB3* mutation p.T383Ifs*13 [31] but was also found to have two novel missense variations in the *USH2A* gene. Sanger sequencing failed to detect one of such *USH2A* variants (thus representing a false negative result) while the presence of the homozygous mutation in *CNGB3* was confirmed.

The remaining 2 index patients from families RP-0886 and RP-0461 were not identified with pathogenic RD mutations. They were therefore all considered as patients for whom molecular diagnosis could not be achieved in absence of additional information.

## Discussion

Because of the elevated genetic and allelic heterogeneity displayed by hereditary retinal degenerations, molecular diagnoses are in general a rather complicated task. Although precise clinical information or family history can facilitate this procedure, the number of known mutations that medical geneticists have to consider in the screening process is likely in the range of a few thousands, over more than 100 different genes. For example, approximately 600 variants/mutations in the *USH2A* gene alone have been reported so far (the USH2A mutations database: http://www.lovd.nl/USH2A). Furthermore, novel RD mutations are constantly being discovered, reducing the value of semi-automated analyses interrogating a specific set of DNA variants.

Starting from the evidence that costs of whole-exome sequencing are constantly lowering and that this technique, although not too sensitive, allows an unsupervised analysis of all of the coding sequences of the human genome, we reasoned that WES could in fact be used in routine DNA-based diagnosis of RD. Our results show an 83% success rate, over 12 families with seemingly recessive RD. This high success rate can partially be explained by the nature itself of recessive conditions, for which two mutations have to be present in the same gene to cause disease. This event alone enables a drastic reduction of the noise associated with WES, since the likelihood that two false negative results (e.g. real DNA changes with no pathogenic effects or simple sequencing errors) affect by chance the same gene is very low. In dominantly inherited cases, for which such a filter cannot be applied, there would be *a priori* no such a way of distinguishing pathogenic variants form rare DNA changes or sequencing false calls. However, although with lower efficiency, WES could in principle still be applied. Important success factors in dominant investigations would be represented by the detection of clear-cut mutations, such as indels and nonsense variants, in known disease genes and the use of control population data analyzed with WES, in order to systematically subtract the noise and enhance the signal throughout the genome. Additionally, the identification of less striking but previously-reported pathogenic changes would also suffice to provide molecular diagnoses to dominant RD cases. For instance, we could efficiently detect a novel frameshift mutation in the *CHM* gene in a male patient with choroideremia. Since *CHM* is located on the chromosome X, this patient and his affected brother do in fact carry a single causative mutation, as in individuals affected by autosomal dominant conditions. Isolated RD cases would be, in the vast majority of the circumstances, either unrecognized recessive cases or *de novo* dominant cases, and therefore could be analyzed by following the same procedures.

NGS has been applied previously to screen for specific forms of RD, with variable results [32]–[34]. For example, NGS provided genetic diagnosis in 36% (36 out of 100) of patients with retinitis pigmentosa [33]. After correcting for the mutation detection rate of NGS and by taking into consideration previously solved cases, the diagnostic yield increased to ∼50%. The comparatively favorable results of the current study that identified 83% of the causes of RD may be attributable to a few specific elements, including the size of the cohort analyzed, the methods and extensiveness of the genetic screenings carried out in the past, the clinical phenotype of the patients, and/or the sequencing/mapping methods of NGS itself.

Another element of note is that out of the 15 mutations identified, 8 (∼50%) were novel, despite the genes in which they were identified were extensively screened for mutations in other cohorts of patients. This finding confirms, unfortunately, the limited value of mutation-based microarrays or sequencing procedures (such as pyrosequencing) in the context of genetics of RD. We also found that wide phenotypic variability of RD and limited clinical information could preclude accurate clinical diagnosis that is essential for current targeted screening methods. In this context, the “screen all RD genes” approach has certainly benefitted three of our cases, initially diagnosed as undefined RD (patient 10/1367), LCA (patient 04/0834), or RP (patient 07/0360), but having mutations in *CNGB3* and *CHM* causing achromatopsia and choroideremia, respectively, that would be missed by ARRP or LCA genotyping microarray. Following such findings, these patients were re-evaluated clinically, and confirmed to have achromatopsia and choroideremia.

Despite the power of WES, validation by Sanger sequencing and, if possible, of additional genetic investigation in family members of the patient remain key elements for the success of this screening technique. The value of familial analyses is particularly evident when we consider the two families for which a DNA diagnosis could not be reached. In these pedigrees, the number of candidate mutations in non-reported RD genes was simply too elevated and, in absence of an additional filtering based on co-segregation of disease and DNA changes in affected relatives, could not be reduced. Moreover, due to limitations that are intrinsic to the exome sequencing procedure, our analyses were underpowered to score DNA copy number variations (CNVs). It is therefore possible that these unsolved cases may in fact carry pathogenic CNVs in RD genes that we were unable to detect.

Methodologically, the procedures used were in general rather simple. We outsourced the sequencing to a large sequencing company, which provided us not only with the raw sequencing reads, but also with an annotated list of variants. These services represent nowadays a standard offer of many private sequencing centers as well as of internal genetic facilities, since most WES protocols and downstream analyses can be streamlined to what closely resembles routine procedures. Computer-based data analysis was also not particularly complicated; we mostly used simple text-parsing scripts and/or commercial spreadsheet programs with filtering capabilities, on common desktop computers.

In our screening we could also gain a few elements of information concerning the molecular pathology of RD mutations. Specifically, we could confirm that missense mutations in *USH2A*, a gene found to be mutated in patients suffering from the blindness-deafness disease known as Usher syndrome, cause in general retinitis pigmentosa without hearing loss (patient 08–177) [35]. We also confirmed that *RP1*, a gene that has been for a long time associated with dominant retinitis pigmentosa, can in fact carry recessive mutations, as previously reported [22], [36]–[42].

In summary, in our work we show that whole-exome sequencing could represent a useful tool for molecular diagnosis of hereditary retinal degeneration, a disease for which standard screening procedures still struggle to produce information in a cost- or time-efficient manner. Further reduction in prices and improvement in sensitivity and specificity, already in progress, would probably make of WES the technique of choice for all future routine DNA diagnoses.

## Supporting Information

Table S1 **PCR primers used in this study.**
(PDF)

Table S2 **Clinical findings of patients.**
(XLSX)

Table S3 **Number of variants identified in patients after each filtering process.**
(PDF)
