# Correction: Exome Sequencing of Index Patients with Retinal Dystrophies as a Tool for Molecular Diagnosis

**DOI:** 10.1371/journal.pone.0153121

**Published:** 2016-03-31

**Authors:** Marta Corton, Koji M. Nishiguchi, Almudena Avila-Fernández, Konstantinos Nikopoulos, Rosa Riveiro-Alvarez, Sorina D. Tatu, Carmen Ayuso, Carlo Rivolta

There are two errors in the RP-1164 row and Nucleotide Change and Protein Change columns of Table 1. The mutation c.863dupA should be c.862dupA and the mutation p.M289Y*18 should be p.T288Nfs*19. There is also an error in Fig 1 under the panel RP-1164. The mutation p.M289Y*18 should be p.T288Nfs*19. Please view the correct Table 1 and Fig 1 here.

**Table 1:** RD mutations identified by WES analyses.

FAMILY ID	INDEX PATIENT ID	GENE (OMIM entry)	NUCLEOTIDE CHANGE	PROTEIN CHANGE	NOVEL/KNOWN	REFERENCE
RP-0674	01–0570	*ABCA4*	c.287delA	p.N96Tfs*19	novel	
		(601691)	c.6148G>C	p.V2050L	known	[43]
RP-0298	95–0103	*ABCA4*	c.4720G>T	p.E1574*	known	[44]
			c.950delG	p.G317Afs*57	novel	
RP-1102	07–0366	*ABCA4*	c.2285C>A (homoz)	p.A762E	known	[45]
RP-1164	07–0360	*CHM* (300390)	c.862dupA	p.T288Nfs*19	novel	
RP-1263	08–0177	*USH2A*	c.920_923dupGCCA	p.H308Qfs*16	known	[46]
		(608400)	c.12574C>T	p.R4192C	novel	
RP-1659	10–1367	*CNGB3*	c.1148delC	p.T383Ifs*13	known	[31]
		(605080)	c.1666G>T	p.E556*	novel	
RP-1174	04–0834	*CNGB3*	c.1148delC (homoz)	p.T383Ifs*13	known	[31]
RP-0137	1601	*RP1*	c.1625C>G	p.S542*	novel	
		(603937)	c.4804C>T	p.Q1602*	novel	
RP-0235	2343	*RP1*	c.5173C>T (homoz)	p.Q1725*	novel	
RP-1116	06–1075	*NMNAT1*	c.507G>A	p.W169*	known	[47]
		(608700)	c.769G>A	p.E257K	known	[47]

**Fig 1:**
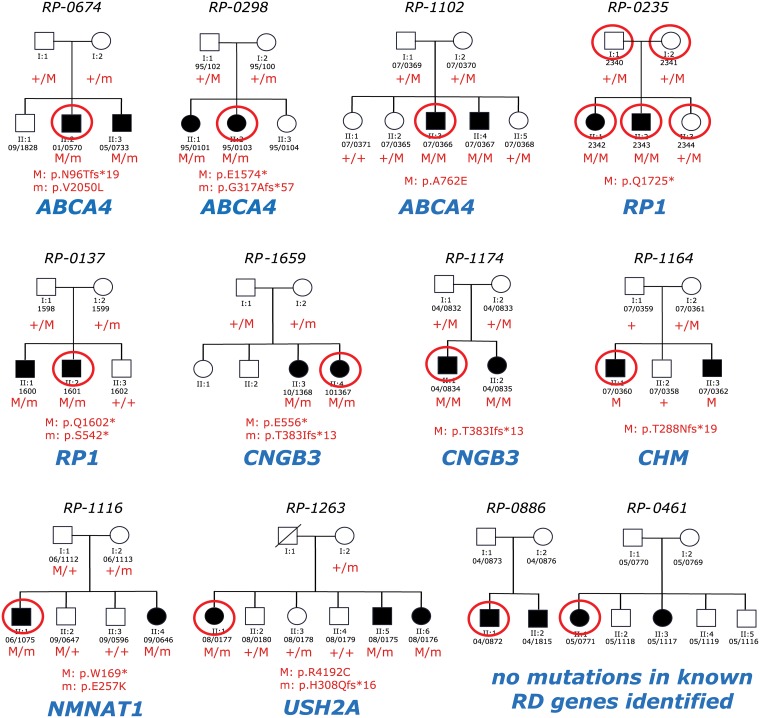
Pedigrees of patients analyzed and mutations identified in this work. The family ID is given above the pedigree, while the individuals’ IDs are indicated below the symbols depicting them. Red circles indicate individuals whose DNA underwent WES analysis. The name of the RD gene identified as causative of the disease is given in blue. M/M, homozygous mutation; M/m compound heterozygous mutations.

## References

[1] Corton M, Nishiguchi KM, Avila-Fernández A, Nikopoulos K, Riveiro-Alvarez R, Tatu SD, et al (2013) Exome Sequencing of Index Patients with Retinal Dystrophies as a Tool for Molecular Diagnosis. PLoS ONE 8(6): e65574 doi: 10.1371/journal.pone.0065574 23940504 10.1371/journal.pone.0065574 PMC3683009

