# Supplementary material for: Exome Sequencing of Index Patients with Retinal Dystrophies as a Tool for Molecular Diagnosis
Source: PLoS One. 2013 Jun 14;8(6):e65574. doi: 10.1371/journal.pone.0065574 (PMC3683009; doi:10.1371/journal.pone.0065574)
Supplement: Table S1 — PCR primers used in this study. (PDF) [file pone.0065574.s001.pdf]

Supplementary Table S1. PCR primers used in this study

Exon	Forward Primer (5'-3')	Reverse Primer (5'-3')	PCR Annealing Temperature (ºC)	Product Size (bp)
ABCA4				
Ex 3	CCTGCTTGGTCTCCATGAC	ACGTGAAGGGGTGTGCAAC	57	249
Ex 8	GAGCATTGGCCTCACAGCAG	CCCCAGGTTTGGTTTCACC	54	400
Ex 15	AGGCTGGTGGGAGAGAGC	GGACTGCTACGGACCATTC	56	373
Ex 33	TTCATGTTTCCCTACAAAACCC	AAAATCCTACTCAAATCTCCAG	58	228
Ex 45	CTTGTCTTCTCCAAATGGCA	TTTAAGCCCTTGGTGCGGC	51	208
CHM				
Ex 7	AGTTATATCATTAGGAAGCAG	TTGGAGAGCACTACTTAATG	55	203
CNGB3				
Ex 10	AAGGGGTTCGCTGGTGAC	CATTGAATGGGTTATGACAGC	60	330
Ex 15	TTTCACATGATATGTTCAACCC	CTTTTCCTTAGGTCGCTTTGG	60	119
NMNAT1				
Ex 5.1	AAAGTAAACCCCTTTCCACTTG	ACCAAGTAGCGAATGCTCTG	58	358
Ex 5.2	TATGAATCGGATGTGCTGTG	TGTGCCCAGACGATTTTTAC	54	384
RP1				
Ex 4.3	CTGTGATTGGCAGTGTGACC	TGCATCTGCTGAAATAGGACTG	60	425
Ex 4.12	CATTTCAGAATTGGAATCTTTTG	CTCTTGGGTAAGTTCGCCAC	60	478
Ex 4.14	CAGGAATTCCAGGAGGAAAG	CAGGTTTCATTGCGAACATC	60	446
USH2A				
Ex 11	TGGCAGGTAGAGATGAAAGG	GCAAATGCAGTCTTCAATTCATC	55	371
Ex 52	TGGGAAGCTGCAAAACTG	GGCCTCAAAGTATGATGGAATG	55	564
