# Supplementary material for: Exome Sequencing of Index Patients with Retinal Dystrophies as a Tool for Molecular Diagnosis
Source: PLoS One. 2013 Jun 14;8(6):e65574. doi: 10.1371/journal.pone.0065574 (PMC3683009; doi:10.1371/journal.pone.0065574)
Supplement: Table S3 — Number of variants identified in patients after each filtering process. (PDF) [file pone.0065574.s003.pdf]

Supplementary Table S3. Number of variants identified in patients after each filtering process

Family ID	Patient ID	Pre-filtering<br>Total variants	Step 1<br>Nonsynonymous	Step 2<br>In 160 RD genes	Step 3<br>MAF < 2%*	Step 4<br>2 Alleles†	Step 5<br>Annotation
RP-0674	01/0570	66145	12223	142	24	9	2
RP-0298	95/0103	67509	12238	112	27	6	2
RP-1102	07/0366	63727	11866	143	26	10	1
RP-0235	2343	70262	12154	108	34	12	1
RP-0137	1601	64148	12021	124	18	8	2
RP1659	10/1367	69980	12559	125	28	8	2
RP-1174	04/0834	67727	12147	124	25	10	4
RP-1164	07/0360	64333	12119	142	28	7	1
RP-1116	06/1075	67962	12110	143	32	13	2
RP-1263	08/08177	67370	12390	112	21	2	2
RP-0886	04/0872	67814	12139	108	21	3	0
RP-0461	05/0771	64652	11935	125	21	9	0
Average		66802	12158	126	25	8.1	1.6
Standard deviation		2211	187	14	4.8	3.3	1.1

Numbers of unique variations in the genome, regardless of their number of copies, are presented (both a homozygous and a heterozygous DNA variant at a given genomic position and of a given type is considered as a single variation).

* Annotation is based on dbSNP build 130.

† For a male patient, a single mutant allele on the X choromosome is also included.
